# Pediatric Dental Emergency Visits and Treatment during Lockdown in the COVID-19 Pandemic: A Retrospective Study

**DOI:** 10.3390/ijerph19073774

**Published:** 2022-03-22

**Authors:** Amir Elalouf, Rubanenko Moran, Bernstein Yaron, Michal Oman

**Affiliations:** 1 Department of Management, Bar-Ilan University, Ramat Gan 5290002, Israel; 2 Dental Research Unit-Maccabi-Dent, Maccabi Healthcare Fund, Tel Aviv 6971028, Israel; moran42@gmail.com (R.M.); bernst_y@maccabi-dent.com (B.Y.); michaloman@gmail.com (M.O.); 3 Department of Pediatric Dentistry, Faculty of Medicine, School of Dentistry, Tel Aviv University, Tel Aviv 6997801, Israel

**Keywords:** COVID-19 pandemic, dental procedures, lockdown period, pediatric patients, treatment

## Abstract

The COVID-19 pandemic has affected all the professions of life, particularly the healthcare sector. In dentistry, prevention of viral spread among healthcare professionals and patients was a substantial challenge. The virus can directly or indirectly infect dentists during dental procedures. This study focuses on the purpose of pediatric patients’ emergency visits to dental clinics and the treatments practiced during the lockdown. It compares the purpose of emergency pediatric patient visits in dental clinics and their treatments before, during, and after the lockdown periods. Computerized data for two consecutive years (2019 and 2020), between 19 March and 30 April and after the lockdown period from 1 May to 12 June 2020, were collected from five random dental clinics in Israel. The emergency visits of patients under 18 years before, during, and after the lockdown was organized into demographic characteristics, the purpose of the visits, and the treatments with medication or dental procedures. Categorical variables were compared and correlated with the chi-squared test and Pearson’s test, respectively, by using SPSS version 21. During the lockdown, emergency cases got appointments for a physical checkup. Herein, we found higher cases of emergency visits of pediatric patients with pain (*n* = 281, 32.6%) than trauma (*n* = 18, 24.7%), followed by infection (*n* = 31, 28.4%) and treatment continuation (*n* = 7, 20.6%) during the lockdown period, compared to before and after the lockdown periods. The patients treated with medication and dental procedures during lockdown were significantly different (*p* < 0.001) than before and after the lockdown. Extraction (*n* = 81, 41.5%), filling (*n* = 84, 50.6%), fluoride (*n* = 13, 92.9%), and pulp treatment (*n* = 92, 42.6%) were substantially practiced in pediatric patients during the lockdown. Further, this study confirmed the rapid adaptation of professional dentists to deal with non-vaccinated pediatric patients and reinforced the better preparation and requirements for such challenges in the future.

## 1. Introduction

The World Health Organization (WHO) published an updateable COVID-19 interactive timeline that confirmed the viral pneumonia outbreak at the end of December 2019 in Wuhan, China [1]. The WHO declared a COVID-19 global pandemic on 11 March 2020, when millions of people were globally infected with the SARS-CoV-2 (severe acute respiratory syndrome coronavirus 2) virus [2]. In Israel, the first coronavirus-infected case was reported on 21 February 2020, and at 15 January 2022, there were 1.72 million infected individuals and 8298 deaths confirmed [3,4,5]. As the cases increased, the Israeli government and the MOH (Ministry of Health) declared a national emergency on 11 March 2020. They enforced various restrictions such as social distancing, closure of flights, and limitations on health services, except for emergencies, including the dental clinics [6]. The global spread of this virus has raised health concerns in the international community. In this context, the unusual restrictions have brought a massive change in social behavior and routine activities, leading to significant stress for the population and medical professions [7].

SARS-CoV-2 is a zoonotic infection abundant in salivary secretions and mucous membranes of the nasopharyngeal and eyes of the infected individuals. The virus significantly spreads through respiratory droplets [8]. The stress and concerns of the virus spreading have also increased among students and dental professionals while performing dental procedures [7]. It was a substantial challenge for the dental professionals to act diligently to prevent the nosocomial spread of the virus among healthcare providers and patients, particularly non-vaccinated pediatric patients [8,9]. The virus might be spread via direct or indirect contacts. During dental procedures, infected patient droplets might be directly transmitted to the dental professionals and indirectly contaminate instruments and surroundings, thus enhancing the risk of virus spread [10,11].

Dental clinics might be the contiguous source of the virus. The American Dental Association (ADA), National Health Commission of China, National Health Service of the United Kingdom, and other worldwide dental associations restricted regular dental procedures and allowed only emergency cases during the lockdown [12,13,14,15]. The emergency case was defined as “potentially life-threatening conditions that require immediate treatment to stop ongoing tissue bleeding and/or alleviate severe pain and/or infection including trauma, cellulitis, and uncontrolled bleeding” [16]. In the case of pediatric patients, exceptional hygiene protocols were required due to non-vaccination. Although, a study confirmed the lesser contribution of children (25%) in spreading COVID-19 compared to adults (44%) [6].

Interestingly, no study has confirmed coronavirus transmission during dental procedures, so most dental care centers were closed due to the fear of transmission. Only a few clinics were allowed to practice while upholding proper safety protocols and hygiene measures [17,18]. However, different reports confirmed the deaths of dentists, dental nurses, and staff due to coronavirus during the pandemic [17,19,20]. Related studies have reported the risks of coronavirus diffusion, along with the recommendations to manage the coronavirus diffusion during dental procedures [21,22,23]. King’s College London and Imperial College London researchers reported an aerosol generation procedure to limit the virus spread and improve safety [24].

To our best knowledge, there are limited reports on the dental procedures performed during the pandemic. A study reported a 90% decrease in dental patients during the lockdown and most patients were treated with extraction surgeries (22.1%), restoration (8.4%), and orthodontic treatments (0.2%) [15]. Kumar et al. reported an increase in dental emergency visits due to abscess, cellulitis, periapical lesions, pulpal, and trauma. Patients were treated with medication and extraction [25]. Nandlal and his colleagues indicated a shift in the pediatric dental treatment from permanent restoration to extraction, pulpectomies, and temporary restoration with the progress to sequela during the lockdown [26]. Liu et al. evaluated Wuhan City children’s dental caries, halitosis, and toothache during the lockdown. Wuhan children were more active in oral hygiene because of regular teeth brushing than the other cities’ children [27]. A retrospective study in Wuhan reported a decrease in dental visits during the lockdown; however, there was an increase in cases under 6 years old. The significant purposes of these dental visits during the lockdown were due to fissures, deep pits, malocclusion, retained primary teeth, and caries. In turn, during treatment, aerosol-generating procedures were less practiced during this period [28]. This study aims to identify the purpose of pediatric patients’ visits to dental clinics and the treatments practiced during the lockdown. The paper also compares and correlates the visit purposes of emergency pediatric patients and the treatment procedures practiced in dental clinics before, during, and after the lockdown in Israel. This study subsequently compares the demographic characteristics with the emergency purposes of visits and the treatment procedures before, during, and after the lockdown periods.

## 2. Methods

### 2.1. Study Design

#### 2.1.1. Selection Criteria and Telemedicine Services

This retrospective study focused on the purpose of visits by and treatments of pediatric patients who visited the Maccabi-Dent in an emergency before, during, and after the lockdown periods. Maccabi-Dent is Israel’s largest first-class Health Maintenance Organizations (HMOs) chain for dental services. It has 53 dental clinics with about 1700 employees spread around the country and serving a 650,000 registered population. After the announcement of lockdown during the COVID-19 pandemic, only 27 clinics were allowed to treat emergency cases. Patients could not attend the clinic without booking an appointment during and after the lockdown (i.e., the appointment was booked by phone). Though, it was not a prerequisite to book an appointment before the lockdown. In parallel, telemedicine and video consultancy services were introduced during the lockdown to facilitate the patients. The telemedicine services helped the patients and dental clinics to handle the non-emergency cases during the lockdown. Only emergency cases got the appointment during the lockdown period. All the patients with severe dental complications and who required urgent oral examination were considered emergency cases. All the other cases were categorized into non-emergency cases. Dentists determined the emergency status of the patients during the telemedicine services that need proper oral examination and treatment similar to the reported study [29]. Sometimes, live video calls or pictures were also used for the proper identification of emergency cases.

**Figure 1:**
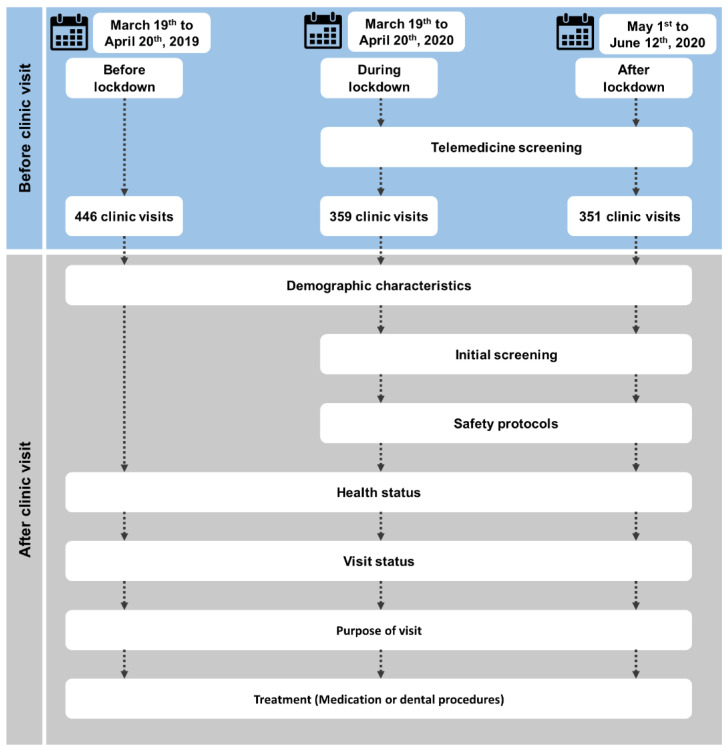
Study design.

Moreover, patients with fever or other coronavirus symptoms did not get an appointment during the lockdown. Patients who got appointments during and after the lockdown underwent an initial screening such as body temperature, breathing problems, and sore throat. After that, safety protocols such as the use of sanitizers, gloves, face masks, and social distancing were strictly followed (Figure 1).

#### 2.1.2. Data Collection

We randomly collected computerized recorded data from the five Maccabi-Dent clinics for two consecutive years (2019 and 2020) between 19 March and 30 April and after the lockdown period from 1 May to 12 June 2020. The data referred to before the lockdown, during the lockdown, and after the lockdown periods in Israel. Figure 1 depicts the step-wise study design. The study was approved by the Ethical Committee of Maccabee Healthcare Services. We arranged the data of patients under 18 years of age to three different periods according to their gender, age groups, health status, visit status, visit purpose, and treatment by medication or dental procedures. The pediatric patients were also categorized into healthy and systematic disease patients in health status and emergency and non-emergency cases in visit status. Before the lockdown, systematic disease patients were also treated in an emergency; thus, this study included such cases. During and after the lockdown, no systematic disease patients, such as those with coronavirus symptoms, got appointments for the physical checkup in the clinic. Pediatric patients’ visits were categorized into trauma, infection, pain, follow-up treatment, and defective treatment in this context.

**Figure 2:**
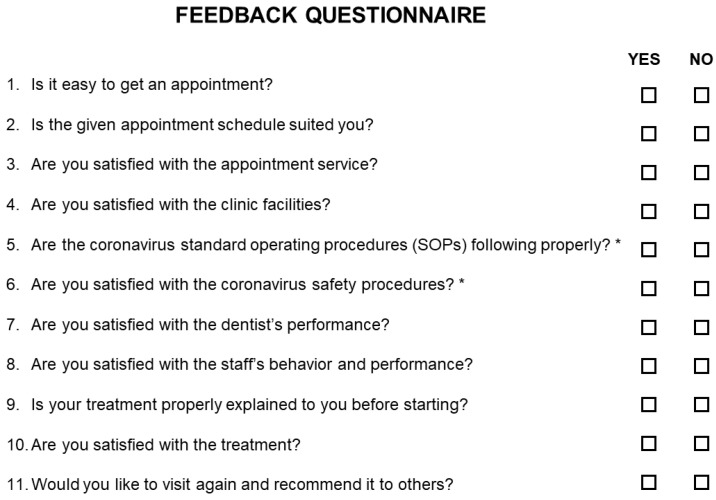
Feedback questionnaire (* the question refers to groups 2 and 3).

Further, pediatric patients were treated with medication or dental procedures. The followed dental procedures were categorized into eight groups. Different procedures were practiced in different periods. Lastly, services feedback was evaluated to improve future services and standard operating procedures (SOPs) in the pandemic. The feedback form (Figure 2) was asked to be filled in by the patients or their caretakers after the treatment.

### 2.2. Statistical Analysis

The data were arranged before lockdown (19 March to 30 April 2019), during lockdown (19 March to 30 April 2020), and after lockdown (1 May to 12 June 2020) in a Microsoft Excel spreadsheet. The frequencies, percentages, means, standard deviations, and ranges were calculated in SPSS version 21; thus, the descriptive statistics. For the inferential statistics, the chi-square test compared the categorical variables of the demographic characteristics with the purpose of visits and treatment procedures by assuming significance at *p* ≤ 0.05. Bonferroni corrections were conducted for the pairwise comparison. In addition, the chi-squared test compared and Pearson’s test correlated the purpose of the visits and treatment procedures before, during, and after the lockdown periods.

## 3. Results

### 3.1. Demographic Characteristics

**Figure 3:**
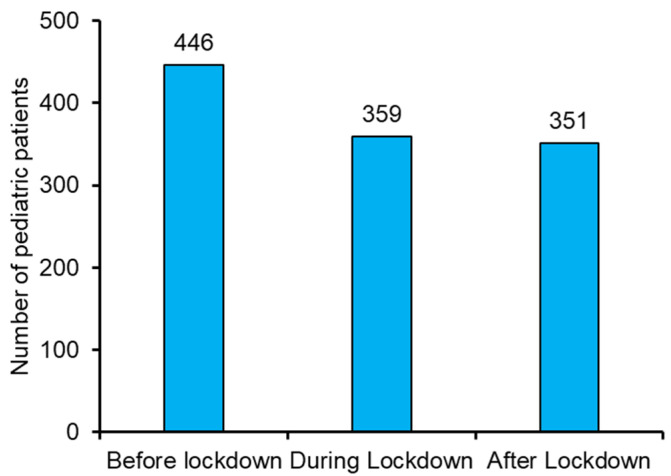
The number of pediatric patient visits in the dental clinics before, during, and after the lockdown.

A total of 359 pediatric patients aged 1–18 years visited five randomly selected dental clinics (Maccabi-Dent) around the country during the lockdown. The total number of pediatric patients visited before, during, and after the lockdown are presented in Figure 3. Table 1 exhibits the demographic characteristics of the three groups included in this study. The three groups were compared based on gender and age by chi-squared test. The comparison for males in three groups was found statistically significant, χ^2^ = 7.436, *p* < 0.01. At the same time, no significant difference was found among the females of all three groups. According to the age categories, all the three groups of pediatric patients were significantly compared with each other, shown in Table 1.

### 3.2. Health and Visit Status

**Figure 4:**
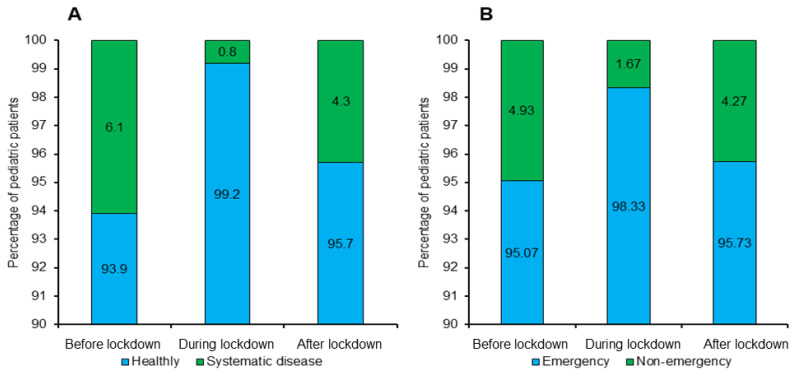
The percentages of pediatric patients’ (**A**) health status and (**B**) visit status before, during, and after the lockdown.

Figure 4 showcases the percentages of health and visit status of the pediatric patients of all the groups. During the lockdown period, healthy patients were only allowed to visit the clinics. Hence, the visits of systematic disease patients were reduced to 0.8%. Systematic disease patients were allowed to visit the clinics before and after the lockdown; therefore, their percentages were higher at those times (Figure 4A). In contrast to patient health, emergency cases were only handled during the lockdown period. As a result, we found more emergency cases (98.33%) during the lockdown than before and after the lockdown (Figure 4B).

### 3.3. Purpose of Visits

All the due appointments were canceled during the lockdown, and only emergency cases were treated. Therefore, this study considered emergency cases of pediatric patients. Table 2 compares and correlates pediatric patients’ purpose of visit before, during, and after the lockdown. The chi-square test significantly stresses the purpose of patients visits due to trauma (χ^2^ (2) = 9.89, *p* < 0.01), infection (χ^2^ (2) = 9.101, *p* = 0.01), and defective treatment (χ^2^ (2) = 10.167, *p* < 0.01) during the lockdown and before and after the lockdown. At the same time, the significant correlation between trauma and infection (r^2^ = 0.855, *p* < 0.001) and treatment continuation and defective treatment (r^2^ = 0.861, *p* < 0.001) were indicated by Pearson’s test. Table 3 shows the comparison between the demographic characteristics and purpose of the emergency visits of all three groups. The purposes of emergency visits of pediatric patients were categorized into five groups: trauma, infection, pain, follow-up treatment, and inadequate treatments. The pain was a significant reason for clinic visits before lockdown in all the age groups and healthy patients. Meanwhile, visit purposes before the lockdown were significantly different regarding age groups (χ^2^ (8) = 185.18, *p* < 0.001), health status (χ^2^ (4) = 121.13, *p* < 0.001), and treatment (χ^2^ (4) = 38.58, *p* < 0.001). In contrast, treatment was only statistically significant (χ^2^ (4) = 26.5, *p* < 0.001) regarding the purpose of visits during the lockdown period. In parallel, medication and treatment procedures regarding infection and pain were substantially different during the lockdown. Lastly, age groups were found statistically significant (χ^2^ (8) = 126, *p* < 0.001) regarding the visit purposes after the lockdown. Interestingly, the purpose of visit during the lockdown was significantly correlated with before (r^2^ = 0.687, *p* < 0.001) and after (r^2^ = 0.923, *p* < 0.001) the lockdown periods.

### 3.4. Treatment

#### 3.4.1. Medication

**Figure 5:**
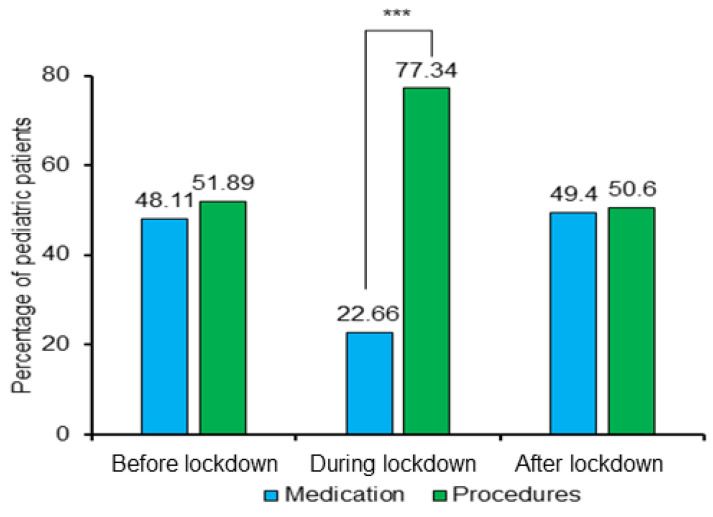
The percentages of pediatric patients treated with medication or dental procedures before, during, and after the lockdown (*** statistically significance at *p* < 0.001).

After the examination, dentists treated the pediatric patients either with medication or recommended a procedure. Figure 5 shows the percentages of pediatric patients treated with medication or dental procedures before, during, and after the lockdown. Before and after the lockdown, the percentages of medical procedures and medicines were almost the same. In turn, the percentage of patients treated with medication had substantially decreased (χ^2^ (1) = 97.407, *p* < 0.001) compared to dental procedures during the lockdown. Moreover, the patients treated with medication during the lockdown had a positive correlation (r^2^ = 0.11, *p* < 0.05) with before the lockdown and a negative correlation (r^2^ = −0.109, *p* < 0.05) with after the lockdown period.

#### 3.4.2. Procedures

Table 4 presents the comparisons and their significance regarding the procedures during the lockdown, namely, crowning (χ^2^ (2) = 30.657, *p* < 0.001), extraction (χ^2^ (2) = 11.108, *p* < 0.01), filling (χ^2^ (2) = 23.181, *p* < 0.001), fluoride (χ^2^ = 10.286, *p* = 0.001), and pulp treatment (χ^2^ (2) = 9.33, *p* < 0.01), regarding before and after the lockdown periods. Moreover, Pearson’s correlation confirmed the significant positive correlations in procedures between crowns and fillings (r^2^ = 0.833, *p* < 0.001), extraction and filling (r^2^ = 0.737, *p* < 0.001), filling and pulp treatment (r^2^ = 0.711, *p* < 0.001), and pulp treatment and extraction (r^2^ = 0.95, *p* < 0.001). Additionally, the emergency cases recommended for the dental procedures were compared with the demographic characteristics and purpose of visits (Table 5). During the lockdown period, the filling dental procedure was statistically significant regarding patients’ visits due to infection. At the same time, the comparison was insignificant between dental procedures and demographic characteristics for the visit purpose before and during the lockdown. However, age categories (χ^2^ (10) = 22.628, *p* = 0.01) and purpose of visit (χ^2^ (20) = 43.943, *p* < 0.01) were significantly different regarding the dental procedures after the lockdown. Further, dental procedures such as extraction, filling, and pulp treatment were more practiced during the lockdown period. In contrast, sealant, space maintainer, and tooth fixation were not practiced in pediatric patients during the lockdown. Moreover, patients who got dental procedures during the lockdown were negatively correlated (r^2^ = −0.043) with before the lockdown and positively correlated (r^2^ = 0.057) with after the lockdown period.

### 3.5. Feedback

**Figure 6:**
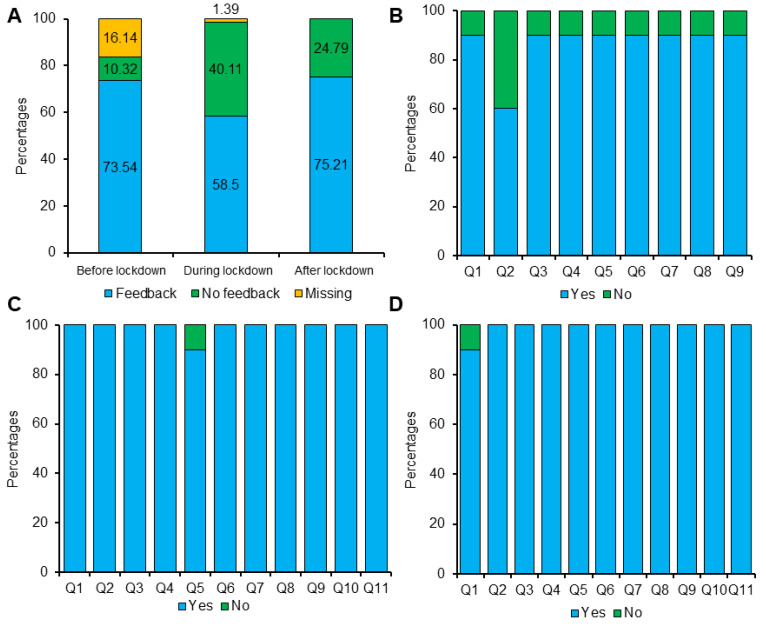
Feedback from the patient: (**A**) available and missing feedback; (**B**–**D**) the percentages of patient feedback before (**B**), during (**C**), and after (**D**) the lockdown, respectively.

After the treatment, patients or their guardians were provided with a feedback form about the services. Figure 6 illustrates the feedback responses. Computerized data were checked for feedback responses. Feedback responses were lower during the lockdown than before and after the lockdown (Figure 6A). Before the lockdown, questions related to SOPs were not present in the feedback forms. Altogether, patients were more satisfied in getting appointments according to their schedule and clinic facilities during the lockdown. In parallel, patients were satisfied with following the SOPs and COVID-19 safety procedures. Interestingly, patients were satisfied with the doctor’s and staff’s performance, behavior, and treatment during and after the lockdown.

## 4. Discussion

This study shows the purpose of dental clinic visits and treatments regarding pediatric patients during the lockdown period. Due to the restrictions endorsed by the Centers for Disease Control and Prevention (CDC) in the dental clinics [30], the emergency cases of pediatric patients were more treated with extraction, filling, and pulp treatment (Table 4). Hence, dental practitioners practiced these treatments to save tooth decay during the lockdown in this context. Meanwhile, a study has reported the risk of dental infections and decay during the pandemic and suggested dental care and hygienic habits to the children [31]. Therefore, this study represents the requirement of proper pediatric dental care during the pandemic, similar to the study of Wuhan children [32], where dental complications among the children were low due to oral health education and knowledge with promoting an attitude of dental care.

The COVID-19 pandemic has substantially intensified the public’s panic and fear of death. Due to limited literature, this study adds scientific data for the future to handle similar situations in medical fields, particularly in dentistry. In the dental clinics, all the appointments were scheduled after a brief interview on the telephone. A dentist decided whether the case was an emergency or not. The interview was mandatory during the pandemic period; therefore, patients found it challenging to get the appointment. Telemedicine services assisted non-emergency cases similar to the related studies [33,34]. Yet, patients were examined for the coronavirus symptoms, such as fever, before entering the clinics.

Interestingly, no COVID-19-positive case was reported in our staff and patients during and after the lockdown period. The dental clinics’ staff thoroughly followed the SOPs; that is why no COVID-19 case was reported. Patients’ feedback on coronavirus SOPs also proved that the protective services were standardized (Figure 6).

The dental professionals are usually in direct contact with the patient’s saliva, nasal, eye, and oral droplets, which require appropriate conscientiousness [35,36]. Thereby, the CDC allowed emergency cases with proper care to prevent the virus from spreading during the lockdown period [30]. A dentist determined the emergency case through telephone or video call. This study shows an increase in pediatric emergency cases (98.33%) during the lockdown (Figure 4B). Nonetheless, in China [37] and Turkey [29], a 38% reduction in pediatric dental emergency cases was reported. Other studies in Israel also reported a decrease in dental emergency cases [35,38]. A total of 20.8% of dental emergency cases were treated in South India [36]. Related studies confirmed an increase in emergency cases during the lockdown. For instance, 68.6% of dental emergency procedures in Nepal [39] and a 29.7% increase in dental emergency visits in China were reported [40].

During the lockdown, the purposes of pediatric patients’ visits in the clinic due to trauma, infection, and treatment continuation were reduced (Table 2). Similar to the studies by Üstün et al. [29] and Fux-Noy et al. [35], herein, the patients’ visits due to pain were higher during the lockdown. Another study reported many trauma cases in males and acute pericoronitis and acute gingivitis cases in females during the lockdown [40]. Notably, we found higher percentages of patients suffering from pain in both genders and ages 7–12 during lockdown (Table 3). Of the patients with pain, 22.66% got medication, while others went through a dental procedure in lockdown (Table 3 and Figure 5). Most cases treated with medication benefited from the telemedicine service, limiting the clinic visits during the lockdown, as reported in related studies [41].

Dental procedures such as extraction, filling, fluoride, and pulp treatment were significantly higher during the lockdown (Table 4). For example, a study reported higher dental procedures of extractions, pulp extirpation, and pulpectomies during lockdown [35]. Another study stated 51.8% of patients were treated with extraction [39]. Related studies also confirmed the practice of extraction procedures during the lockdown [5,29]. In addition, filling (17.88%) was more practiced in males, and pulp treatment (17.51%) was more practiced in females during the lockdown. Similarly, patients with pain got the dental procedure of pulp treatment (25.91%) and filling (24.08%), mentioned in Table 5. In addition, feedback confirmed the patients’ satisfaction towards treatment, dentists’ performance, and safety protocols during the pandemic.

This study mainly focuses on pediatric patients’ visits due to emergencies in different periods. However, further studies must fill the study gaps related to the adult population, non-emergency cases, prescribed medication, medication with dental procedures, dental professionals, and patients’ perceptions of telemedicine and virtual examination. Such studies will contribute to the scientific literature to handle challenging situations in the future.

Overall, the study confirms the dentists’ professional behavior and quick adaptation to non-vaccinated pediatric patients in health emergencies. In addition, this study reinforces the better preparation and handling of such situations in the future.

## 5. Conclusions

The global COVID-19 has influenced emergency cases for healthcare procedures, particularly in dentistry. Only emergency cases with no systematic disease symptoms were allowed to visit the clinics during the lockdown. Most pediatric visits due to pain and infection were treated with dental extraction, filling, and pulp treatment during the lockdown. However, this study is limited to emergency visits of pediatric patients during the lockdown. It is also significant that all the patients were non-vaccinated children, and no coronavirus-positive case was reported in patients and staff during the lockdown. This study provides a direction for better preparation in the future to handle such emergency situations in dentistry.

## Figures and Tables

**Table 1:** Demographic data.

Demographic Characteristics	Before Lockdown *n* = 446 (100%)	During Lockdown *n* = 359 (100%)	After Lockdown *n* = 351 (100%)	*p*-Value
**Gender**				
**Male**	234 (52.5)	178 (49.58)	187 (53.28)	<0.01
**Female**	212 (47.5)	181 (50.42)	164 (46.72)	0.879
**Age**				
**Range**	1–17	1–18	1–16	
**Mean + SD**	7.9 + 4.16	8.51+ 4.18	7.14 + 2.62	
**Median**	7	8	7	
**≤6**	156 (34.98)	111 (30.92)	110 (31.34)	<0.001
**7–12**	241 (54.04)	161 (44.85)	172 (49)	<0.001
**13–18**	49 (10.99)	87 (24.23)	69 (19.66)	<0.01

**Table 2:** Comparison and correlation among the purpose of the pediatric patients’ emergency visits before, during, and after the lockdown.

Visit Purposes	Before Lockdown	During Lockdown	After Lockdown	*p*-Value	Pearson’s Correlation
**1 Trauma, *n* = 73, (100%)**	37 (50.7)	18 (24.7)	18 (24.7)	<0.01	1 vs. 2 (*p* < 0.001)
**2 Infection, *n* = 109, (100%)**	51 (46.8)	31 (28.4)	27 (24.8)	0.01	
**3 Pain, *n* = 861, (100%)**	307 (35.7)	281 (32.6)	273 (31.7)	0.33	
**4 Treatment continuation, *n* = 34, (100%)**	12 (35.3)	7 (20.6)	15 (44.1)	0.23	4 vs. 5 (*p* < 0.001)
**5 Defective treatment, *n* = 36, (100%)**	17 (47.2)	16 (44.4)	3 (8.3)	<0.01	

**Table 3:** Comparison between the demographic characteristics and purpose of the pediatric patient’s emergency visits before, during, and after the lockdown.

Demographic Characteristics	Trauma *n* (%)	Infection *n* (%)	Pain *n* (%)	Treatment Continuation *n* (%)	Defective Treatment *n* (%)	*p*-Value
**Before lockdown, *n* = 424, (100%)**						
**Gender**						
**Male**	12 (2.83)	24 (5.66)	169 (39.85)	8 (1.89)	10 (2.35)	0.07
**Female**	25 (5.9)	27 (6.36)	138 (32.54)	4 (0.94)	7 (1.65)	
**Age groups**						
**≤6**		5 (1.18) ^a^	150 (35.37) ^a^			<0.001
**7–12**	30 (7.07) ^a^	43 (10.14) ^a^	146 (34.43) ^a^	1 (0.23) ^a^	12 (2.83)	
**13–18**	7 (1.65) ^a^	3 (0.7)	11 (2.6) ^a^	11 (2.6) ^a^	5 (1.18) ^a^	
**Health Status**						
**Healthy**	37 (8.72)	51 (12.03)	307 (72.4) ^a^	12 (2.83)	12 (2.83) ^a^	<0.001
**Systemic Disease**					5 (1.18) ^a^	
**Treatment**						
**Medication**	21 (4.95)	16 (3.77)	167 (39.38)			<0.001
**Procedures**	16 (3.77)	35 (8.25)	140 (33.01)	12 (2.7)	17 (3.81)	
**During lockdown, *n* = 353, (100%)**						
**Gender**						
**Male**	10 (2.83)	11 (3.11)	142 (40.22)	3 (0.85)	10 (2.83)	0.405
**Female**	8 (2.26)	20 (5.66)	139 (39.37)	4 (1.13)	6 (1.7)	
**Age groups**						
**≤6**	4 (1.13)	12 (3.4)	85 (24.08)		7 (2.00)	0.409
**7–12**	11 (3.11)	13 (3.68)	125 (35.41)	4 (1.13)	7 (2.00)	
**13–18**	3 (0.8)	6 (1.7)	71 (20.11)	3 (0.85)	2 (0.56)	
**Health Status**						
**Healthy**	18 (5.1)	31 (8.78)	281 (79.6)	7 (2.00)	16 (4.53)	
**Treatment**						
**Medication**			80 (22.66) ^a^			<0.001
**Procedures**	18 (5.1)	31 (8.78) ^a^	201 (56.94) ^a^	7 (2.00)	16 (4.53)	
**After lockdown, *n* = 336, (100%)**						
**Gender**						
**Male**	11 (3.27)	15 (4.46)	143 (42.56)	7 (2.08)	1 (0.3)	0.865
**Female**	7 (2.08)	12 (3.57)	130 (38.7)	8 (2.38)	2 (0.6)	
**Age groups**						
**≤6**		2 (0.6)	86 (25.5)	15 (4.46) ^a^	1 (0.3)	<0.001
**7–12**	13 (3.86)	2 (0.6) ^a^	152 (45.23) ^a^		1 (0.3)	
**13–18**	5 (1.49)	23 (6.84) ^a^	35 (10.41) ^a^		1 (0.3)	
**Health Status**						
**Healthy**	18 (5.35)	27 (8.03)	273 (81.25)	15 (4.46)	3 (0.9)	
**Treatment**						
**Medication**	11 (3.27)	18 (5.35)	129 (38.39)	7 (2.08)		0.104
**Procedures**	7 (2.08)	9 (2.67)	144 (42.85)	8 (2.38)	3 (0.9)	

^a^ Bonferroni correction shows a statistically significant pairwise comparison at *p* < 0.05.

**Table 4:** Comparison and correlation among the treatment procedures of pediatric patients before, during, and after the lockdown.

Treatment Procedures	Before Lockdown	During Lockdown	After Lockdown	*p*-Value	Pearson’s Correlation
**1 Crown, *n* = 67, (100%)**	41 (61.2)	4 (6)	22(32.8)	<0.001	1 vs. 3 (*p* < 0.001)
**2 Extraction, *n* = 195, (100%)**	70 (35.9)	81 (41.5)	44 (22.6)	<0.01	2 vs. 3 (*p* < 0.001)
**3 Filling, *n* = 166, (100%)**	36 (21.7)	84 (50.6)	46 (27.7)	<0.001	3 vs. 5 (*p* < 0.001)
**4 Fluoride, *n* = 14, (100%)**	1 (7.1)	13 (92.9)		0.001	
**5 Pulp treatment, *n* = 216, (100%)**	68 (31.5)	92 (42.6)	56 (25.9)	<0.01	5 vs. 2 (*p* < 0.001)
**6 Sealant, *n* = 2, (100%)**	2 (100)				
**7 Space maintainers, *n* = 3, (100%)**	2 (66.7)		1 (33.3)	0.564	
**8 Tooth fixation, *n* = 2, (100%)**			2 (100)		

**Table 5:** Comparison between the demographic characteristics and dental procedures of pediatric patients’ emergency visits before, during, and after the lockdown.

Demographic Characteristics	Crown *n* (%)	Extraction *n* (%)	Filling *n* (%)	Fluoride *n* (%)	Pulp Treatment *n* (%)	Sealant *n* (%)	Space Maintainer *n* (%)	Tooth Fixation *n* (%)	*p*-Value
**Before lockdown, *n* = 220, (100%)**									
**Gender**									
**Male**	25 (11.36)	37 (16.81)	15 (6.81)		35 (15.9)	1 (0.45)	1 (0.45)		0.68
**Female**	16 (7.27)	33 (15)	21 (9.54)	1 (0.45)	33 (15)	1 (0.45)	1 (0.45)		
**Age**									
**≤6**	15 (6.81)	22 (10)	6 (2.72)		16 (7.27)	1 (0.45)	1 (0.45)		0.776
**7–12**	23 (10.45)	38 (17.27)	26 (11.81)	1 (0.45)	43 (19.54)	1 (0.45)	1 (0.45)		
**13–18**	3 (1.36)	10 (4.54)	4 (1.81)		9 (4.09)				
**Health status**									
**Healthy**	40 (18.18)	69 (31.36)	35 (15.9)	1 (0.45)	66 (30)	2 (0.9)	2 (0.9)		0.998
**Systemic Disease**	1 (0.45)	1 (0.45)	1 (0.45)		2 (0.9)				
**Purpose of visits**									
**Trauma**	1 (0.45)	7 (3.18)	2 (0.9)		6 (2.72)				0.98
**Infection**	6 (2.72)	11 (5)	6 (2.72)		12 (5.45)				
**Pain**	31 (14.09)	40 (18.18)	23 (10.45)	1 (0.45)	41 (18.63)	2 (0.9)	2 (0.9)		
**Treatment continuation**	1 (0.45)	7 (3.18)	1 (0.45)		3 (1.36)				
**Defective Treatment**	2 (0.9)	5 (2.27)	4 (1.81)		6 (2.72)				
**During lockdown, *n* = 274, (100%)**									
**Gender**									
**Male**	2 (0.73)	35 (12.77)	49 (17.88)	7 (2.55)	44 (16.06)				0.429
**Female**	2 (0.73)	45 (16.42)	35 (12.77)	6 (2.19)	48 (17.51)				
**Age**									
**≤6**	1 (0.36)	24 (8.76)	33 (12.04)	5 (1.82)	20 (7.3)				0.066
**7–12**	3 (1.1)	40 (14.6)	34 (12.4)	2 (0.73)	49 (17.88)				
**13–18**		16 (5.84)	17 (6.2)	6 (2.19)	23 (8.4)				
**Health status**									
**Healthy**	4 (1.46)	80 (29.19)	84 (30.65)	13 (4.74)	92 (33.57)				
**Purpose of visits**									
**Trauma**		7 (2.55)	5 (1.82)		6 (2.19)				0.12
**Infection**		12 (4.38)	8 (3.00) ^a^	5 (1.82)	6 (2.19)				
**Pain**	4 (1.46)	54 (19.7)	66 (24.08)	6 (2.19)	71 (25.91)				
**Treatment continuation**		3 (1.1)			4 (1.46)				
**Defective Treatment**		4 (1.46)	5 (1.82)	2 (0.73)	5 (1.82)				
**After lockdown, *n* = 171, (100%)**									
**Gender**									
**Male**	11 (6.43)	21 (12.28)	25 (14.62)		32 (18.71)			1 (0.58)	0.865
**Female**	11 (6.43)	23 (13.45)	21 (12.28)		24 (14.03)		1 (0.58)	1 (0.58)	
**Age**									
**≤6**	9 (5.26)	14 (8.18)	17 (9.94)		22 (12.86)				0.012
**7–12**	11 (6.43)	25 (14.61)	24 (14.03)		28 (16.37)				
**13–18**	2 (1.17)	5 (2.92)	5 (2.92)		6 (3.5)		1 (0.58)	2 (1.17)	
**Health status**									
**Healthy**	22 (12.86)	44 (25.73)	46 (26.9)		56 (32.74)		1 (0.58)	2 (1.17)	
**Purpose of visits**									
**Trauma**		2 (1.17)	3 (1.75)		2 (1.17)				0.002
**Infection**	1 (0.58)	3 (1.75)	1 (0.58)		2 (1.17)			2 (1.17)	
**Pain**	19 (11.1)	36 (21.05)	40 (23.4)		48 (28.07)		1 (0.58)		
**Treatment continuation**	2 (1.17)	1 (0.58)	2 (1.17)		3 (1.75)				
**Defective Treatment**		2 (1.17)			1 (0.58)				

^a^ Bonferroni correction shows a statically significant pairwise comparison at *p* < 0.05.

## Data Availability

Supporting data is not required.
